# Association of Regular Leisure-Time Physical Activity with Self-Reported Body Mass Index and Obesity Risk among Middle-Aged and Older Adults in Taiwan

**DOI:** 10.3390/healthcare9121719

**Published:** 2021-12-13

**Authors:** Yun-Tsung Chen, Po-Fu Lee, Chi-Fang Lin, Andy Chang, Yu-Chun Chung, Chi-Pei Yeh, Shih-Chang Chen, Chien-Chang Ho

**Affiliations:** 1 Department of Physical Education and Sport Sciences, National Taiwan Normal University, Taipei City 106, Taiwan; cthero178@hotmail.com (Y.-T.C.); aa830521@gmail.com (C.-F.L.); 2 Department of Leisure Industry and Health Promotion, National Ilan University, Yilan 260, Taiwan; pflee@niu.edu.tw (P.-F.L.); ncc5207@niu.edu.tw (S.-C.C.); 3 Air Permits Division, Texas Commission on Environmental Quality, P.O. Box 13087, Austin, TX 78711, USA; andy.chang@tceq.texas.gov; 4 Center of General Education, Taipei Medical University, Taipei 11031, Taiwan; e750224@tmu.edu.tw; 5 Department of Physical Education, Fu Jen Catholic University, New Taipei City 24205, Taiwan; S37081915@gmail.com; 6 Research and Development Center for Physical Education, Health, and Information Technology, College of Education, Fu Jen Catholic University, New Taipei City 24205, Taiwan; 7 Artificial Intelligence Development Center, Fu Jen Catholic University, New Taipei City 24205, Taiwan

**Keywords:** physical activity, obese, underweight, health status

## Abstract

Through this study, we aimed to determine the association of regular leisure-time physical activity (LTPA) with self-reported body mass index (BMI) and obesity risk among middle-aged and older adults in Taiwan. We conducted a cross-sectional study and reviewed the data derived from the Taiwan National Physical Activity Survey (TNPAS). Responses from 12,687 participants aged 45–108 years from the database were collected in this study. All the participants completed a standardized structured questionnaire that solicitated information regarding their demographic characteristics (age, gender, education, occupation, and self-reported health status), physical activity behaviors (regular/nonregular LTPA), and self-reported anthropometrics (height, weight, and BMI). Multiple linear and logistic regressions were used to examine the association between regular LTPA and BMI, and between regular LTPA and obesity status, respectively. Regular LTPA was associated with male gender, normal weight, excellent or good self-reported health status, and a lower rate of being underweight compared with nonregular LTPA. Regular LTPA was significant negatively associated with being underweight (OR = 0.71, *p* < 0.05), whereas it had no significant relationship with BMI and obesity (*p* > 0.05). Regular LTPA was associated with a reduced risk of being underweight among middle-aged and elderly adults in Taiwan. Further research on the relevant mechanism underlying this phenomenon is warranted.

## 1. Introduction

According to the World Health Organization (WHO), the global prevalence of obesity has nearly tripled since 1975, and approximately 13% of adults worldwide (over 650 million) were classified as obese in 2016 [1]. A person with obesity is defined as having a body mass index (BMI) greater than or equal to 30 kg/m^2^, and it may increase the risk of developing coronary heart disease, hypertension, type 2 diabetes, sleep apnea, osteoarthritis, and some cancers [2]. However, Gurrici et al. suggested that the cut-off point for obesity based on BMI in Asian individuals should be 27 kg/m^2^, as Asian individuals of the same age and gender with the same body fat percentage have a lower BMI by approximately 3 kg/m^2^ than Caucasian individuals [3].

It has been reported that one’s obesity status is associated with socioeconomic status and family and environmental factors [4,5]. For example, the greatest number of obese adults live in highly developed countries with higher incomes (e.g., Austria and the UK), which results in a sedentary lifestyle and changes in eating habits, such as a diet rich in highly processed foods and fast foods, as well as excess energy intake [6]. Children who have an overweight mother, or a family history of obesity, or who come from a single-parent family may have increased access to high-caloric foods and less recreational exercise, thus are at increased risk of overweight and obesity [7]. In addition, the availability of neighborhood sidewalks, parks, and recreational facilities has been reported to facilitate physical activity (PA) and decrease the prevalence of obesity [4].

PA is recommended as an important part of weight management to enhance weight loss and prevent weight regain [8]. Moreover, PA is associated with a decreased risk of cardiovascular disease (CVD), stroke, diabetes, metabolic syndrome, and cancer (e.g., colon and breast cancer) [8,9]. However, high-income countries exhibit significantly lower PA compared to low-income countries [6]. The American College of Sports Medicine (ACSM) recommends that overweight or obese adults should perform moderate-intensity PA for ≥150 min/week to improve their health, and progressively increase to 200–300 min/week for long-term weight loss [10]. However, 31% of adults (especially older adults) worldwide are physically inactive, leading to approximately 3 million deaths per year and costing global healthcare systems approximately USD 50 billion [11,12].

Age-induced changes in body fat percentage and BMI increase the risk of obesity, cardiometabolic diseases, and disability [13]. According to the WHO, the terms “aging society” and “aged society” refer to societies with ≥7% and ≥14% of individuals aged ≥65 years, respectively [14]. Taiwan was considered an aging society in 1993 and became an aged society in 2018 [15]. It is estimated that, in 2026, Taiwan will enter the era of a “super-aged society”, when the proportion of elderly adults could be ≥20% [16]. Therefore, successful aging is an important issue for individuals in Taiwan to reduce the risk of chronic disease, maintain physical and cognitive function, and sustain active engagement with life [17].

Leisure-time physical activity (LTPA) refers to PA such as sports, exercise, and recreational walking or cycling undertaken during leisure time [18]. Numerous studies have found that older adults who participate in LTPA have a reduced risk of many chronic diseases and increased physical and cognitive function, as well as improved quality of life [18,19]. Although Taiwan is a highly technologically developed country, to our knowledge, no study has yet reported the relationship between LTPA and BMI and obesity risk considering the health status, education, and occupation of middle-aged and elderly adults. Therefore, through the present study, we aimed to investigate the association of regular LTPA with self-reported BMI and obesity risk, and identify the determining factors that influence this relationship among middle-aged and older adults in Taiwan.

## 2. Materials and Methods

### 2.1. Study Design and Participants

We performed a cross-sectional observational study using nationally representative data regarding middle-aged and older adults (age range 45–108 years) from the Taiwan National Physical Activity Survey (TNPAS) conducted by the Taiwan Sports Administration, Ministry of Education. Recruitment was conducted through random-digit dialing of phone numbers of a sample of the population proportionally stratified by age, gender, and geographical district; the detailed procedure has been described elsewhere [20]. The participants were citizens older than 13 years of age, stratified by 22 cities/counties across Taiwan. The sample size of each city/county was calculated by its proportion to the population of the country. The total sample size was 25,526 in 2020, with sampling errors equal to or less than 5% and a confidence interval (CI) of 95%, which provided sufficient sample size and statistical power. Subsequently, a computer-assisted telephone interview (CATI) survey was conducted from August to October 2020. Well-trained and experienced interviewers were employed for the CATI survey to ensure the quality of data collection. Data collected from the telephone survey questionnaire included information on sociodemographic characteristics (age, gender, education, occupation, and zip code of residence), LTPA behaviors, and self-evaluations (including health status and anthropometric variables (height and body weight)). Finally, 12,687 participants were enrolled in this study. The study was conducted in accordance with the Declaration of Helsinki, and all the procedures were in accordance with the ethical standards of the Institutional Review Board of Fu Jen Catholic University in Taiwan (FJU- IRB C109085). Oral consent was provided by the participants before telephone interviews.

### 2.2. Data Collection

Sociodemographic characteristics including age, gender, education, occupation, and zip code of residence; LTPA behaviors; and self-evaluations on health status and anthropometric variables (height and body weight) of the participants were recorded. The participants were divided into five groups according to their age (45–49, 50–54, 55–59, 60–64, and ≥65 years). Education was categorized into three levels (elementary school or lower, junior or senior high school, and college or higher). Occupation was divided into 11 categories (white collar, government servant, blue collar, owner/manager, specialist, student, housewife, retired, freelancer, jobless, and other). Self-reported health status was categorized into three levels (excellent or good, fair, and very bad or poor).

### 2.3. Anthropometric Variables and Obesity Status

Anthropometric variables including height and body weight were obtained from the CATI survey, and BMI (kg/m^2^) was further calculated. Then, BMI cut-off points were adopted as suggested by the Health Promotion Administration, Ministry of Health and Welfare in Taiwan: underweight (BMI < 18.5 kg/m^2^), normal weight (BMI between 18.5 and 24 kg/m^2^), overweight (BMI between 24 and 27 kg/m^2^), and obese (BMI ≥ 27 kg/m^2^) [3,21].

### 2.4. LTPA Assessment

The LTPA of participants was assessed by a series of questions during the CATI survey. First, participants were asked to describe their current LTPA participation by answering the question “Have you participated in any LTPA in the past month?” Respondents who responded “yes” were then asked to note the frequency and duration of their participation, with questions such as “How many times do you participate in LTPA per week?” and “How many minutes do you usually spend at one time?” Further, the intensity of LTPA was assessed based on the breathing and sweating status of participants with the question “When you are doing LTPA, you usually feel?” The participants chose a response from structural answers such as “No changes in my breath and sweating”, “I breathe faster but do not sweat”, “I breathe normally but sweat”, and “I breathe quickly and sweat”. Participants who reported that they usually breathed quickly and sweated were thus considered to engage in moderate-intensity LTPA. Participants were then dichotomized into regular and non-regular LTPA groups according to the physical activity (PA) recommendations specified in WHO guidelines. Participants who reported performing ≥150 min of moderate-intensity LTPA or ≥75 min of vigorous-intensity LTPA per week and responded “yes” to breathing quickly and sweating were considered the regular LTPA group, and the rest as the non-regular LTPA group.

### 2.5. Statistical Analysis

Data were analyzed using SAS 9.4 (SAS Institute, Cary, NC, USA). The normal distribution of data was examined by using the Shapiro–Wilk test. Student’s t-test was performed to analyze continuous variables, and the chi-square test was used to analyze categorical variables. Multiple linear regression analysis with BMI as the dependent variable was used to examine the association of regular LTPA with BMI after adjusting for potential confounders. Adjusted odds ratios (ORs) with 95% CI for people with obesity, overweight, or underweight were calculated from unconditional logistic regression models according to regular LTPA. Values are presented as mean ± standard deviation or frequency percentage. Results were considered two-tailed and statistically significant at *p* < 0.05.

## 3. Results

A total of 12,687 participants were dichotomized into regular and non-regular LTPA groups based on their LTPA status. Their sociodemographic characteristics and anthropometric variables are presented in Table 1. All the participants were divided into either the regular or non-regular LTPA group; the non-regular LTPA group had a larger proportion (79%) of participants. Except for BMI (*p* = 0.063), significant differences were observed between the groups (*p* < 0.05) in terms of all the relevant variables, including age, gender, height, body weight, BMI, obesity status, education, occupation, and self-reported health status (*p* < 0.05). The regular LTPA group had a higher proportion of males (53.3%) and normal-weight individuals (48.8%) and a higher education rate (27% of participants had completed college education); almost 34.9% of participants had already retired, and 75.5% self-reported that they were in excellent or good health.

Table 2 presents the results of a comparison of BMI values between regular and non-regular LTPA status among middle-aged and older adults. Pooled data show significant differences (*p* < 0.05) in the group of oldest participants (≥65 years). Participants in the regular LTPA group had higher BMI values than those in the other group (24.01 ± 2.91 kg/m^2^). In addition, BMI values differed significantly between regular and non-regular LTPA groups among the youngest male participants (age 45–49 years; *p* < 0.05). Men in the regular LTPA group had higher BMI values than those in the other group (25.39 ± 3.22 kg/m^2^).

The results of the comparison between the prevalence of obesity and LTPA status among middle-aged and older adults are shown in Table 3. With regard to obesity status, significant differences between regular and non-regular LTPA groups were found only in the underweight group in pooled data (*p* < 0.05) from the group of participants aged 50–54 years. A higher prevalence (4.00%) of being underweight was observed in the non-regular LTPA group. After separating men and women, significant differences were observed between the two underweight groups among participants aged 45–49 years, and the non-regular LTPA group had a higher prevalence of being underweight (men, 1.10%; women, 5.40%). Moreover, among participants aged 50–54 years, the prevalence of being underweight significantly differed between the two groups; the non-regular LTPA group had a higher prevalence of being underweight (1.60%).

Table 4 presents the results of multivariate regression for regular LTPA and BMI scores, indicating a positive relationship (*β* = 0.021, *p* < 0.05) in Model 1. More regular LTPA translated to higher BMI scores. However, after adjusting for age, gender, self-reported health status, occupation, and education, no significant differences were found between regular LTPA and BMI scores (*p* = 0.108).

The results of multivariate logistic regression for regular LTPA and obesity, overweight, and underweight risk are shown in Table 5. Irrespective of adjustment for age, gender, self-reported health status, occupation, and education, a significant and negative relationship was observed between regular LTPA and being underweight (Model 1: OR = 0.638, Model 2: OR = 0.713, *p* < 0.05).

## 4. Discussion

To our knowledge, this is the first study to recruit middle-aged and older adults without regular LTPA by considering variety in gender, obesity status, health status, education, and occupation factors in Taiwan. The main findings of this study are as follows: (1) regular LTPA was associated with a reduced risk of being underweight among middle-aged and older adults; and (2) a higher proportion of adults who performed regular LTPA had normal weight and excellent or good self-reported health status, and there was a lower rate of being underweight among these adults compared with those with non-regular LTPA. These findings suggest that regular LTPA may maintain or promote a toned figure and perceived good health status, and reduce the risk of being underweight among middle-aged and elderly adults.

The association of demographic variables such as age, gender, income, ethnicity, and residential location with PA has been reported [22]. For example, the proportion of physical inactivity is higher in women than in men (40% vs. 28%), and physical inactivity is more common in high-income countries than in low-income countries (45% vs. 25%), and this is more prevalent in the Americas than in Southeast Asia (43% vs. 17%) [12]. People who live in highly developed countries with a higher income can make significant changes to their eating habits, such as consuming a diet rich in simple sugars, animal fats, and highly processed foods, and exceed the recommended daily intake of energy, and therefore may be at increased risk of obesity [6]. Furthermore, the prevalence of obesity was higher in Uyghur participants and lower in Muslim Chinese when compared with Han participants, which may result from differences in cultures, dietary habits, and economic levels among the different ethnic populations in China [23]. In the present study, regular LTPA was associated with male sex (53%), normal weight (49%), college education (27%), and being retired (35%), and 76% of the participants self-reported excellent or good health status. A similar study indicated that elderly people with a high level of education are more predisposed to having a healthy lifestyle and dietary habits, thereby reducing their risk of obesity [24]. In addition, the prevalence of obesity was shown to increase among Korean men with higher monthly income or education level, which may be attributed to a sedentary lifestyle, a high-calorie diet, and frequent alcohol intake during working days. By contrast, the risk of obesity was reduced in Korean women with higher monthly income or education level, which may be attributed to the pursuit of a slim body, healthy diet, and active lifestyle [25].

ACSM suggested that both moderate- and vigorous-intensity PA (≥150 and ≥75 min/week, respectively) had beneficial effects on weight management and reduced the risk of chronic diseases, cognitive impairment, disability, and premature mortality [8,9,19]. Hallal et al. reported that adults’ participation in moderate- and vigorous-intensity PA decreased with age [12]. In the present study, we observed that only 21% of middle-aged and elderly adults performed regular LTPA in Taiwan. Notably, the proportion of regular LTPA among adults increased with age, from 16–18% among middle-aged adults to 33% among elderly adults. A similar study reported that PA levels generally decrease with age, whereas an improvement in regular PA was observed as adults reached retirement age (≥65 years) [26]. A significant reduction in PA has been reported in highly technologically-developed countries with increased use of computers, televisions, and cell phones, which encourages a more sedentary lifestyle [6]. On the other hand, living in a highly developed culture with sports facilities or fitness clubs, including outdoor gyms, bike paths, and playgrounds, may boost PA levels [6]. Future research should investigate whether an internal or external motivation (e.g., health enhancement, national policy development, economic pressure release, or sociocultural change) promotes regular LTPA by adults at retirement age.

A study reported that BMI was significantly associated with dependency during activities of daily living by elderly individuals and that this dependency decreased with the practice of LTPA. Moreover, no significant interaction was noted between BMI and LTPA with regard to an association with dependency in activities of daily living, which indicates substantial confounding effects between BMI and LTPA [27]. In this study, we found that regular LTPA was associated with healthy BMI among middle-aged and elderly adults, but became nonsignificant after adjusting for confounding factors (age, gender, self-reported health status, occupation, and education). Confounding factors may overestimate or underestimate the real magnitude of an association, or even change the direction of a real association [28]. Therefore, the use of properly adjusted confounding factors is necessary to avoid bias and distortion.

Notably, previous studies have indicated that being underweight was associated with increased all-cause mortality compared with normal weight [29,30]. Such high mortality among individuals that were underweight may be attributable to smoking, preexisting illness, and increased risk of injury and impaired survival after an accident [29]. In the current study, we observed that regular LTPA was associated with a reduced risk of being underweight (BMI < 18.5 kg/m^2^). Compared with non-regular LTPA, regular LTPA was associated with a lower prevalence of being underweight in men aged 45–54 years and women aged 45–49 years. Another study reported a significant correlation between PA and muscle mass among young and older adults [31,32]. Sagiv et al. indicated that regular PA reduced height loss due to aging in both men and women [33]. Therefore, we speculate that regular LTPA may promote muscle mass and bone health and prevent height loss [19,33], resulting in optimized BMI and prevention of becoming underweight among middle-age adults.

The present study has some limitations. First, the health status, LTPA, and BMI assessments were based on self-reported questionnaires and therefore have considerable potential for misclassification [13,26,27,34]. However, the large sample size is the major strength of this study, which provides an idea of how cross-sectional changes occur in LTPA and obesity status among middle-aged and older adults. Secondly, this study did not measure the body fat percentage, muscle mass, and bone mineral density of the participants. Without these measurements, it may be difficult to comprehensively elucidate the mechanisms underlying the reduced risk of being underweight by regular LTPA. Third, our questionnaire did not include the reason for participating in regular LTPA, thus the motivation behind the improved regular LTPA observed among adults at retirement age remains unclear. Finally, due to the cross-sectional nature of this study, no cause and effect relationships can be discerned. Future studies should be conducted with a longitudinal study design in order to elucidate the clinical importance of regular LTPA in reducing underweight risk.

## 5. Conclusions

This study demonstrates that regular LTPA was associated with increased perceived good health status and reduced risk of being underweight among middle-aged and elderly adults. Hoverer, regular LTPA showed no relationship with BMI and obesity after adjusting for confounding factors (e.g., age, gender, and occupation). Further research on the association between regular LTPA, body fat percentage, and bone mineral density in Taiwanese adults is warranted.

## Figures and Tables

**Table 1:** Demographic characteristics of participants.

Variables	LTPA Status		*p*-Value
	Regular LTPA (*n* = 2641)	Non-Regular LTPA (*n* = 10,046)	
Age (y)			0.037 *
45–49	427 (16.20%)	1655 (16.50%)	
50–54	480 (18.20%)	1647 (16.40%)	
55–59	434 (16.40%)	1721 (17.10%)	
60–64	438 (16.60%)	1525 (15.20%)	
≥65	862 (32.60%)	3497 (34.80%)	
Gender (% men)	1407 (53.3%)	4452 (44.3%)	<0.001 *
Height (cm)	163.62 ± 7.78	161.92 ± 7.95	<0.001 *
Body weight (kg)	64.60 ± 11.61	62.88 ± 11.63	<0.001 *
BMI (kg/m^2^)	24.04 ± 3.38	23.90 ± 3.46	0.063
Obesity status (%)			<0.001 *
Underweight	53 (2.00%)	310 (3.10%)	
Normal weight	1289 (48.80%)	4805 (47.80%)	
Overweight	770 (29.20%)	2668 (26.60%)	
Obese	407 (15.40%)	1572 (15.60%)	
Education (%)			<0.001 *
Elementary school or lower	340 (13.00%)	1904 (19.10%)	
Junior or senior school	1570 (60.00%)	6100 (61.30%)	
College or higher	706 (27.00%)	1940 (19.50%)	
Occupation (%)			<0.001 *
White collar	287 (10.90%)	894 (9.00%)	
Government servant	161 (6.10%)	408 (4.10%)	
Blue collar	323 (12.30%)	1931 (19.40%)	
Owner/manager	194 (7.40%)	630 (6.30%)	
Specialists	104 (4.00%)	353 (3.50%)	
Student	0 (0.00%)	2 (0.00%)	
Housewife	433 (16.50%)	2124 (21.40%)	
Retired	917 (34.90%)	2790 (28.00%)	
Freelancer	121 (4.60%)	445 (4.50%)	
Jobless	69 (2.60%)	328 (3.30%)	
Other	15 (0.60%)	42 (0.40%)	
Self-reported health status (%)			<0.001 *
Excellent or good	1967 (77.50%)	6826 (71.90%)	
Fair	184 (7.30%)	902 (9.50%)	
Very bad or poor	386 (15.20%)	1767 (18.60%)	

BMI, body mass index; LTPA, leisure-time physical activity. * *p* < 0.05. Values expressed as mean ± standard deviation for continuous variables.

**Table 2:** Comparison of BMI values according to LTPA status among middle-aged and older adults in Taiwan.

Variables	LTPA Status		*p*-Value
	Regular LTPA (*n* = 2641)	Non-Regular LTPA (*n* = 10,046)	
Men (*n* = 5502)			
45–49	25.39 ± 3.22	24.76 ± 3.16	0.015 *
50–54	24.44 ± 2.65	24.75 ± 3.06	0.140
55–59	24.73 ± 2.84	24.39 ± 3.00	0.136
60–64	24.54 ± 2.44	24.31 ± 2.86	0.267
≥65	24.30 ± 2.59	24.05 ± 2.86	0.073
Women (*n* = 6153)			
45–49	22.43 ± 2.29	22.54 ± 3.06	0.575
50–54	22.79 ± 2.73	23.00 ± 3.11	0.312
55–59	22.71 ± 2.92	23.09 ± 2.96	0.109
60–64	22.99 ± 2.91	23.24 ± 3.01	0.294
≥65	23.57 ± 3.28	23.45 ± 3.03	0.529
Pooled (*n* = 11,655)			
45–49	23.87 ± 3.15	23.52 ± 3.30	0.058
50–54	23.66 ± 2.81	23.72 ± 3.20	0.717
55–59	23.76 ± 3.05	23.66 ± 3.05	0.587
60–64	23.80 ± 2.78	23.73 ± 2.99	0.665
≥65	24.01 ± 2.91	23.74 ± 2.96	0.024 *

BMI, body mass index; LTPA, leisure-time physical activity. * *p* < 0.05.

**Table 3:** Comparison between prevalence of overweight/obesity and LTPA status among middle-aged and older adults in Taiwan.

Variables		LTPA Status		*p*-Value
		Regular LTPA (*n* = 2641)	Non-Regular LTPA (*n* = 10,046)	
Men (*n* = 5502)				
45–49	Underweight	0 (0.00%)	7 (1.10%)	0.004 *
	Normal weight	76 (38.60%)	262 (39.50%)	
	Overweight	47 (23.90%)	221 (33.30%)	
	Obesity	74 (37.60%)	174 (26.20%)	
50–54	Underweight	0 (0.00%)	10 (1.60%)	0.001 *
	Normal weight	98 (40.80%)	232 (37.10%)	
	Overweight	105 (43.80%)	223 (35.60%)	
	Obesity	37 (15.40%)	161 (25.70%)	
55–59	Underweight	3 (1.40%)	13 (1.80%)	0.170
	Normal weight	76 (36.40%)	312 (43.40%)	
	Overweight	90 (43.10%)	251 (34.90%)	
	Obese	40 (19.10%)	143 (19.90%)	
60–64	Underweight	0 (0.00%)	15 (2.20%)	0.127
	Normal weight	100 (45.70%)	294 (44.00%)	
	Overweight	85 (38.80%)	242 (36.20%)	
	Obesity	34 (15.50%)	117 (17.50%)	
≥65	Underweight	3 (0.60%)	35 (2.40%)	0.062
	Normal weight	219 (46.40%)	728 (48.90%)	
	Overweight	177 (37.50%)	505 (33.90%)	
	Obesity	73 (15.50%)	220 (14.80%)	
Women (*n* = 6153)				
45–49	Underweight	11 (5.30%)	45 (5.40%)	0.003 *
	Normal weight	149 (71.30%)	553 (66.00%)	
	Overweight	44 (21.10%)	151 (18.00%)	
	Obese	5 (2.40%)	89 (10.60%)	
50–54	Underweight	6 (2.80%)	52 (5.70%)	0.077
	Normal weight	146 (67.60%)	538 (59.10%)	
	Overweight	45 (20.80%)	215 (23.60%)	
	Obesity	19 (8.80%)	106 (11.60%)	
55–59	Underweight	7 (3.60%)	40 (4.40%)	0.131
	Normal weight	136 (69.00%)	542 (60.20%)	
	Overweight	36 (18.30%)	225 (25.00%)	
	Obesity	18 (9.10%)	93 (10.30%)	
60–64	Underweight	6 (3.00%)	27 (3.50%)	0.704
	Normal weight	125 (63.10%)	460 (59.80%)	
	Overweight	48 (24.20%)	187 (24.30%)	
	Obesity	19 (9.60%)	95 (12.40%)	
≥65	Underweight	17 (5.30%)	63 (4.00%)	0.404
	Normal weight	164 (51.10%)	881 (55.30%)	
	Overweight	93 (29.00%)	449 (28.20%)	
	Obesity	47 (14.60%)	201 (12.60%)	
Pooled (*n* = 11,655)				
45–49	Underweight	11 (2.70%)	52 (3.50%)	0.556
	Normal weight	225 (55.40%)	815 (54.30%)	
	Overweight	91 (22.40%)	372 (24.80%)	
	Obesity	79 (19.50%)	262 (17.50%)	
50–54	Underweight	7 (1.50%)	62 (4.00%)	0.001 *
	Normal weight	243 (53.40%)	769 (50.10%)	
	Overweight	150 (33.00%)	438 (28.50%)	
	Obesity	55 (12.10%)	267 (17.40%)	
55–59	Underweight	10 (2.50%)	53 (3.30%)	0.801
	Normal weight	213 (52.30%)	855 (52.80%)	
	Overweight	126 (31.00%)	476 (29.40%)	
	Obesity	58 (14.30%)	236 (14.60%)	
60–64	Underweight	6 (1.40%)	42 (2.90%)	0.239
	Normal weight	226 (54.20%)	754 (52.50%)	
	Overweight	132 (31.70%)	429 (29.90%)	
	Obesity	53 (12.70%)	212 (14.80%)	
≥65	Underweight	19 (2.40%)	97 (3.10%)	0.104
	Normal weight	383 (48.30%)	1609 (52.20%)	
	Overweight	271 (34.20%)	953 (30.90%)	
	Obesity	120 (15.10%)	421 (13.70%)	

LTPA, leisure-time physical activity. * *p* < 0.05.

**Table 4:** Multivariate regression for regular LTPA and BMI scores.

Variables	Model 1 (Unadjusted)			Model 2 (Adjusted ^a^)		
	*β*	SE	*p*-Value	*β*	SE	*p*-Value
Regular LTPA	0.021	0.069	0.022 *	0.015	0.068	0.108
Non-regular LTPA	Ref.	-	-	Ref.	-	-

LTPA, leisure-time physical activity; BMI: body mass index; SE, standard error. * *p* < 0.05. ^a^ Adjusted for age, gender, self-reported health status, occupation, and education.

**Table 5:** Multivariate logistic regression for regular LTPA and risk of obesity, being overweight, and being underweight.

Variables	Model 1 (Unadjusted)			Model 2 (Adjusted ^a^)		
	*β*	SE	*p*-Value	*β*	SE	*p*-Value
Obesity						
Regular LTPA	1.035	0.913–1.173	0.590	0.985	0.862–1.125	0.822
Non-regular LTPA	Ref.	-	-	Ref.	-	-
Overweight						
Regular LTPA	1.076	0.972–1.190	0.157	1.046	0.941–1.162	0.403
Non-regular LTPA	Ref.	-	-	Ref.	-	-
Underweight						
Regular LTPA	0.638	0.474–0.859	0.003 *	0.713	0.527–0.965	0.028 *
Non-regular LTPA	Ref.	-	-	Ref.	-	-

CI, confidence interval; LTPA, leisure-time physical activity; OR, odds ratio. * *p* < 0.05. ^a^ Adjusted for age, gender, self-reported health status, occupation, and education.

## Data Availability

The data supporting the findings of this study are available from the Sports Cloud: Information and Application Research Center of Sports for All, Sport Administration, Ministry of Education in Taiwan, but restrictions apply to the availability of the data, which were used under license for the current study, and so are not publicly available. However, data are available from the authors upon reasonable request and with permission of the Sports Cloud: Information and Application Research Center of Sports for All, Sport Administration, Ministry of Education in Taiwan.

## Abbreviations

LTPA	Regular leisure-time physical activity
BMI	Body mass index
TNPAS	Taiwan National Physical Activity Survey
WHO	World Health Organization
PA	Physical activity
CVD	Cardiovascular disease
ACSM	American College of Sports Medicine
CI	Confidence interval
CATI	Computer-assisted telephone interview
FJU-IRB	Institutional Review Board of Fu Jen Catholic University
OR	Odds ratio
SE	Standard error
